# Government R&D Subsidies, Environmental Regulations, and Their Effect on Green Innovation Efficiency of Manufacturing Industry: Evidence from the Yangtze River Economic Belt of China

**DOI:** 10.3390/ijerph17041330

**Published:** 2020-02-19

**Authors:** Ming Yi, Yiqian Wang, Modan Yan, Lina Fu, Yao Zhang

**Affiliations:** 1 School of Economics and Management, China University of Geosciences, Wuhan 430074, China; yiming@cug.edu.cn (M.Y.); wangyiqianwang87@gmail.com (Y.W.); zhangyao@cug.edu.cn (Y.Z.); 2 Faculty of Business and Economics, Monash University, Melbourne 3800, Australia; 3 School of International Education, South-central University for Nationalities, Wuhan 430074, China

**Keywords:** government R&D subsidies, environmental regulations, green innovation efficiency of manufacturing industry, panel Tobit model

## Abstract

The Yangtze River Economic Belt is the most important manufacturing economic belt in China. The level of manufacturing green innovation efficiency of the Yangtze River Economic Belt directly affects the overall competitiveness of China’s manufacturing industry. With panel data from 11 provinces and cities along the Yangtze River Economic Belt in China for the period of 2008 to 2017, this paper applies the slacks-based measure (SBM)-data envelopment analysis (DEA) model and panel Tobit model to conduct an empirical study of the effects of government research and development subsidies and environmental regulations on the green innovation efficiency of the manufacturing industry of the Yangtze River Economic Belt. The results show that, firstly, government R&D subsidies and environmental regulations are both conducive to improving the green innovation efficiency of the manufacturing industry of the Yangtze River Economic Belt; secondly, because of the fact that the interaction terms between government R&D subsidies and environmental regulations failed to pass the significance test, the positive moderating effects of R&D subsidies on environmental regulations and green innovation efficiency of the manufacturing industry are not obvious; thirdly, in terms of control variables, strengthening agglomeration is the only factor that is positively correlated with green innovation efficiency improvement of the manufacturing industry. Enterprise scale and industrial structure have negative effects on green innovation efficiency improvement, and the openness of economy has no correlation with green innovation efficiency.

## 1. Introduction

At present, the concept of green innovation has become a hot topic because of a tense practical need for sustainable development. Generally speaking, green innovation is equivalent to ecological innovation, or environmental innovation, among others, and is considered to be an innovative activity involving process, system, and service, through which sustainable development aims can be achieved by reducing environmental damage and natural resource consumption. Green innovation plays a role in achieving sustainable development goals (EIO [1]). In other words, green innovation is not just about new green technology, it covers various kinds of innovations, such as new products, new processes, new services, new business models, and so on (Kemp and Arundel [2]; Rennings [3]; Kemp and Pearson [4]). Accordingly, green innovation of the manufacturing industry can be defined as “green innovation of technology, product, and processes, and corresponding organizational, management, and institutional innovation processes”. It is a systematic and open innovation of behavior and process aiming at green development of the manufacturing industry. From the perspective of input–output, efficiency refers to “the ratio between the input and output of human behavior”. Combined with the definition of manufacturing green innovation, the manufacturing green innovation efficiency can be simply defined as “the ratio between input and output of manufacturing green innovation in a certain period of time”. It is a relative relationship between the input of human, finance, materials, technology, information, and so on, and a certain amount of green innovation output.

The Yangtze River Economic Belt covers 11 provinces and municipalities in eastern, central, and western China. Along the belt, 44% of China’s total industrial output, more than 50% of the total output value of emerging industries, and one-third of China’s universities/research institutions are clustered. Moreover, there are a number of internationally competitive clusters in electronic information, high-end equipment, automobiles, home appliances, textiles and clothing manufacturing, and so on. Therefore, the Yangtze River Economic Belt is considered to be the most strategically supporting and the strongest manufacturing economic belt. However, at the same time, the discharge of industrial sewage and energy consumption account for more than 40% of the total amount in China. Especially, the discharge of chemical oxygen demand, ammonia nitrogen, total nitrogen, and total phosphorus in industrial sewage is close to 50% of the national total. With the economic growth in the Yangtze River Economic Belt, the heavy chemical manufacturing structure also leads to a series of resource and environmental problems. Faced with severe resource and environment constraints and relatively limited innovation resources, how can the manufacturing industry in the Yangtze River Economic Belt achieve more and higher quality green innovation output under certain green innovation input? That is, how to improve the manufacturing green innovation efficiency (hereinafter referred to as “MGIE”)? It is the key scientific and practical problem that needs to be solved for the sustainable development of China’s manufacturing industry.

The driving factors of MGIE are generally discussed from internal and external aspects. Many researchers prefer to use internal factors such as technology push (Horbach [5]), Research and Development (R&D) input (Baumol [6]), governance level (Amore and Bennedsen [7]; Bernauer [8]), application of new enterprise software and new equipment (Demirel and Kesidou [9]), and so on. However, there are many other studies using external variables to discuss the driving factors of MGIE. They are market pull (Horbach [5]), government behavior (Liao [10]), foreign direct investment (Andonova [11]), industrial agglomeration (Carlino et al. [12]), social culture (Huang and Li [13]), pressure of regulators (Huang et al. [14]), and so on. What should be noted is that the externality of green innovation activities, market failure, and path dependence of technological innovation make it more difficult for enterprises to obtain benefits from green innovation activities and improve MGIE (Marin [15]; Rubashkina et al. [16]) to a certain extent. Consequently, it is urgent to improve MGIE with the help of government. As such, government R&D subsidies and environmental regulations are important tools of government intervention. 

First, most scholars believe that government support for R&D activities is conducive to improving MGIE (Hud and Hussinger [17]). R&D subsidies from government can fill the gaps in R&D funds of enterprises, stimulate R&D initiatives because of profit maximization (Seitz and Watzinger [18]), and further promote green innovation behavior (Wang et al. [19]). However, Guan and Chen [20] had a different idea. They found that, compared with corporate capital itself, the role of government funds in improving MGIE is likely to be weaker or even invalid. Wallsten [21] even found that government R&D subsidies had a crowding-out effect on the R&D input within enterprises. As a result, there was a significant negative relationship between government R&D subsidies and MGIE. 

Second, environmental regulations have been regarded as an important variable in investigating MGIE. On the one hand, environmental regulations have a “follow-the-cost effect” (Greenstone [22]). In the short term, the “hard” conditions imposed by the government will increase the cost of green innovation activities, consequently reduce the input of enterprises in green innovation activities, and thus hinder the improvement of MGIE (Christainsen and Haveman [23]; Gray et al. [24]). On the other hand, some scholars point out that environmental regulations have an “innovation compensation effect” (Rio et al. [25]), which implies that the compliance cost incurring from environmental regulations can be shared and MGIE be improved in consequence. The goal of improving the competitiveness of manufacturing enterprises while protecting the environment can be achieved (Simpson and Bradford [26]). However, some scholars think that the relationship between environmental regulations and MGIE is not simply linear. The relationship between the two can be affected by pollution intensity (Li and Tao [27]), regional heterogeneity (Zhang et al. [28]), regulatory tool type (Shen et al. [29]), and other factors.

In general, there are still some deficiencies in the study of the impact of government intervention on MGIE. First, the current studies on government R&D subsidies and environmental regulations on MGIE generally focus on the impact of individual government intervention on MGIE. However, some scholars have noticed that the government R&D subsidies have a regulatory impact on the relationship between environmental regulations and green innovation, but they fail to reach a consistent conclusion. Therefore, this paper introduces the interaction between government R&D subsidies and environmental regulations for further consideration. Second, the non-linear relationship between environmental regulations and MGIE has not been properly considered, which means whether or not a turning point exists needs to be tested. Third, the opposite effects of MGIE on government R&D subsidies and environmental regulations are ignored (Li et al., [30]). If we study the impact of R&D subsidies and environmental regulations on MGIE from only one perspective, endogenous problems may arise, which may have a certain impact on the accuracy of the research and the effectiveness of the policy promulgation and implementation.

Therefore, selecting the Yangtze River Economic Belt of China as the research area, this paper constructs a theoretical analysis framework of the impact of government R&D subsidies and environmental regulations on MGIE. With panel data from 2008 to 2017, taking into consideration of endogenous problems, this paper applies the slacks-based measure (SBM)-data envelopment analysis (DEA) model to measure the level of MGIE in the Yangtze River economic belt, and through the use of panel Tobit model, an empirical test is done to measure the effects of government R&D subsidies and environmental regulations on MGIE in the Yangtze River economic belt. The main contributions of this paper are as follows. First, not only government R&D subsidies, environmental regulations, and the second term of environmental regulations are included in the model, but the interaction term between government R&D subsidies and environmental regulations is considered to evaluate the way government intervention affects MGIE under the combination of the two policies. Second, considering the two-way interaction effect, the instrumental variable method is used to further correct the endogenous problem.

The paper is organized as follows. Section 3 describes the theoretical basis and proposes three research hypotheses. Section 4 outlines the research model. Empirical results and analysis are presented in Section 5. The last section provides a conclusion and several policy implications.

## 2. Theoretical Basis and Research 

### 2.1. Mechanism of Government R&D Subsidies Affecting MGIE

On the one hand, government R&D subsidies may have a positive effect on MGIE. First of all, green innovation activities take a lot of time and resources. During this period, enterprises may face many practical problems, such as changes in market demand, technical competition from competitors, alterations of key technical talents, and insufficient supply of funds. The fact that enterprises themselves bear the huge risks and uncertainties of green innovation activities undoubtedly weakens the enthusiasm to carry out green innovation. Besides, green innovation activities have positive externality. Other enterprises can enjoy the benefit through imitation, replication, and so on. “Hitchhiking” makes it impossible for even the successful R&D enterprises to enjoy all the benefits from green innovation, which causes a serious blow to the enthusiasm for green innovation again. Finally, because of the existence of information asymmetry, the information flow between manufacturing enterprises and external investors is not smooth enough to achieve effective matching between capital supply and demand, which leads to the shortage of funds in the development stage of green technology (Colombo et al. [31]). Government R&D subsidies can motivate enterprises to carry out green innovation. The economic signals released by the government can provide other external financing channels for relevant enterprises (Wu [32]), which is conducive to alleviating the problem of insufficient funds as well as reducing the cost and risk of green technology R&D activities. Through financial support, manufacturing enterprises can also be guided to shift the focus to green innovation. The mode of production will shift from relying on traditional elements such as labor, capital, and energy to focusing on R&D activities. Consequently, the effective utilization of innovation input resources will be improved. On this basis, Hypothesis 1a is proposed as follows.

**Hypothesis** **1a** **(H1a).**  *Government R&D subsidies can improve MGIE.*

Government R&D subsidies may also have a negative effect on MGIE. Because of the information asymmetry, along with the imperfect supervision system of the government, government R&D subsidies may have “crowding out” effect on R&D investment. Driven by the profit-seeking nature of capital, government R&D subsidies are likely to flow to low-end industries with relatively small investment environment cost, high profit level, and low risk (Liu [33]). Government R&D subsidies may also be used to develop production technologies that tend to improve productivity rather than green innovation technologies that can save energy and reduce emissions (Yu et al. [34]). In this case, it is not beneficial to improve the resource and environment, as well as MGIE. On this basis, Hypothesis 1b is proposed as follows.

**Hypothesis** **1b** **(H1b).**  *Government R&D subsidies can inhibit MGIE.*

### 2.2. The Mechanism of Influence of Environmental Regulations on MGIE

On the one hand, there is an “innovation compensation effect” in environmental regulations, and environmental regulations have a positive role on MGIE. All the benefits of innovation can even exceed the total compliance cost. In view of the green development trend, environmental regulations provide an opportunity for enterprises to analyze their current constraints and adapt to the development trend of the market in future (Lanoie [35]). Consequently, a green technological innovation revolution in the whole industrial chain will take place. In the long run, environmental regulations help manufacturing enterprises reduce energy consumption, pollutant emissions, and improve production efficiency, thus improving MGIE (Porter and Van der Linde [36]). On the other hand, there is a “follow-the-cost effect” in environmental regulation, and environmental regulations have a negative effect on MGIE. Because of the negative externalities of technological innovation and the ‘public good’ characteristics of the environment, the environment could easily be polluted, and because of environmental regulations, the social cost of this pollution can be internalized to a certain degree. The stronger the intensity of environmental regulation, the higher the cost. That is to say, in the process of production, the greater the cost of destroying the environment and consuming resources, the more innovation funds are squeezed out, which means, under the condition that other elements remain unchanged, the innovation activities will be restricted, which is not conducive to improving MGIE.

It should be noted that the “innovation compensation effect” and “follow-the-cost effect” are not totally synchronous. In the long term, with the continuous improvement of the environmental regulation system and the intensification of environmental regulations, the cost that enterprises have to pay for violating the environmental regulations is increasing. Enterprises start to recognize the importance of replacing old growth drivers with new ones, and begin to increase investment in innovation, speed up research and development of green technology, and produce environmentally-friendly and resource-saving products. When the “innovation compensation effect” gradually exceeds the “follow-the-cost effect”, the positive role of environmental regulations in MGIE will be highlighted. On this basis, Hypothesis 2a is proposed as follows.

**Hypothesis** **2a** **(H2a).**  *Environmental regulations can improve MGIE.*

However, in the early stage of environmental regulations, the cost of violating environmental regulations is not high because of the weakness of environmental regulations. Enterprises lack the stimulus to carry out green innovation, with the “follow-the-cost effect” being a dominant role during the time. On this basis, Hypothesis 2b is proposed as follows.

**Hypothesis** **2b** **(H2b).**  *Environmental regulations can inhibit MGIE.*

### 2.3. Mechanism of the Joint Effect of R&D Subsidies and Environmental Regulation on MGIE

From a comprehensive perspective, the combined effect of government intervention on MGIE is in line with the current actual needs. It is conducive to the understanding of the complementary mechanism between different policies. On this basis, subsequent relevant policies can be put forward to improve MGIE. The generation and development of green technology innovation, as well as the marketization and industrialization of innovation, depend largely on whether the interactive mechanism between government policies is effective (He [37]). With an effective interaction, government R&D subsidies can reduce the compliance costs through abiding by environmental regulations, and supplementing the R&D investment. In addition, government R&D subsidies can send “signals” to the market, which help bring other external financing opportunities, reduce the financing risk of manufacturing enterprises, and increase innovative output. (Kleer [38]). During this process, government subsidy and environmental regulation can verify and supplement each other, which demonstrates the fact that green innovation is the only path to realize the high-quality transformation of the manufacturing industry; as a result, the new fashion of green manufacturing will be inspired in the whole society. On this basis, Hypothesis 3a is proposed as follows.

**Hypothesis** **3a** **(H3a).**  *Interaction between government R&D subsidies and environmental regulations can improve MGIE.*

However, too much government funding will inhibit social support, which is not conducive to the positive role of voluntary environmental regulations in promoting green innovation (Rooij et al. [39]). Apart from that, because of rent-seeking, market failure, and so on, the green innovation activities led by government may result in inefficient administrative measures, and a strange phenomenon may appear where innovation input keeps growing, but innovation output remains unchanged. On this basis, Hypothesis 3b is proposed as follows.

**Hypothesis** **3b** **(H3b).**  *Interaction between government R&D subsidies and environmental regulations can inhibit MGIE.*

**Figure 1:**
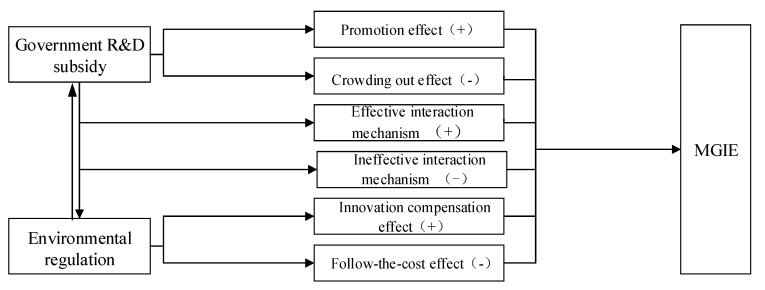
Mechanism of the effects of government Research and Development subsidies and environmental regulation on manufacturing green innovation efficiency (MGIE). Note: ‘−, +’ represent negative and positive correlation, respectively, between R&D subsidies, environmental regulation, the interactive term of the two, and MGIE.

The mechanism of this paper is shown in Figure 1.

## 3. Data and Methods

### 3.1. Model Construction

Because the traditional data envelopment analysis (DEA) model has strict requirements on input and output factors, the non-radial, non-angle SBM-DEA model is proposed on this basis (Tone and Kao [40]). Compared with the stochastic frontier approach (SFA), the SBM-DEA model is more suitable to measure the efficiency with multiple inputs and multiple outputs (Zhou [41]). The calculation model of MGIE can be seen in the Appendix. Accordingly, with the consideration of undesirable outputs, this paper uses the SBM-DEA model to measure MGIE (Liu et al. [42]). On the basis of Honoré [43] and Honoré [44], this paper uses the panel Tobit model to estimate the effect of government R&D subsidies and environmental regulations on MGIE. Besides, the quadratic term of environmental regulations is introduced to explore the non-linear relationship between environmental regulations and MGIE. The definition of panel Tobit models is shown in Formulas (1), (2), (3), and (4), which are recorded as model 1, 2, and 3.

(1) $$MGTE_{it}^{*}=SUB_{it}\zeta +X_{it}\beta +\alpha_{i}+\varepsilon_{it}$$

(2) $$MGTE_{it}^{*}=ER_{it}\psi +ER_{it}^{2}\xi +X_{it}\beta +\alpha_{i}+\varepsilon_{it}$$

(3) $$MGTE_{it}^{*}=SUB_{it}\gamma +ER_{it}\eta +\kappa ER_{it}^{2}+X_{it}\beta +\alpha_{i}+\varepsilon_{it}$$

(4) $$MGTE=\max\{0,MGTE^{*}\}$$

In the above formulas, *i*, t refers to province *i* in year *t*. $MGTE_{it},SUB_{it},ER_{it}$ refer to MGIE, government R&D subsidies, and environmental regulations for province i in year t, respectively. $X_{it},\gamma ,\zeta ,\psi ,\xi ,\gamma ,\eta ,\kappa ,\beta ,\alpha_{i},\varepsilon_{it}$ refer to the control variables, the parameters to be estimated, individual fixed effect, and random effect, respectively.

In order to further analyze the interaction between government R&D subsidies and environmental regulations, and their correlation with MGIE, as shown in Formula (5), the interactive term between government R&D subsidies and environmental regulations is introduced on the basis of Formula (3), and is recorded as model 4, where ω,ξ,ϑ,ζ refer to the parameters to be estimated, respectively.

(5) $$MGTE^{*}=SUB_{it}\omega +ER_{it}\xi +ER_{it}^{2}\vartheta +SUB*ER\zeta +X_{it}\beta +\alpha_{i}+\varepsilon_{it}$$

It should be noted that the effects and significance of government R&D subsidies and environmental regulations on MGIE will not influence the test result of the interactive term. That is to say, only the direction and significance of the interactive term need to be analyzed (Brambor et al. [45]).

### 3.2. Selection of Variables, Source of Data, and Data Processing 

With MGIE as the dependent variable, and based on the assumption of constant returns to scale (CRS), technical efficiency is selected as the proxy variable of MGIE.

#### 3.2.1. Input Variables

Input variables mainly include human input, financial input, and resources (energy) input. (1) R&D personnel is an important component of green innovation in manufacturing industry, and R&D human input plays a decisive role in improving MGIE. Therefore, human input is measured by the full-time equivalent of R&D personnel in industrial enterprises above a designated size. (2) Financial input also has a decisive impact on the efficiency of resource allocation. The increase of financial input directly leads to the increase of innovation activities. Therefore, financial input is measured by the R&D expenditure in industrial enterprises above a designated size. (3) Energy consumption level is closely related to production efficiency (Fadi [46]). In the process of green innovation in the manufacturing industry, a certain amount of resources (energy) input is needed. The less energy consumption per unit of industrial production, the less environmental damage and resource waste, thus improving MGIE. Therefore, resources (energy) input is measured by the energy consumption per unit of industrial production.

#### 3.2.2. Output Variables

Output variables include expected output and unexpected output. On the one hand, patent is an important expected output of green innovation. The number of patents is widely used to measure the creation of new knowledge [47,48]. However, patents cannot reflect the market transformation of innovation at times; therefore, sales revenue of a new product can be used to make up for this problem. Expected outputs are measured by proxy variables as the number of domestic invention patents and sales revenue of a new product in enterprises above a designated size. On the other hand, along with the improvement of production efficiency brought by innovation activities, pollution problems such as industrial sewage, industrial waste gas, industrial solid waste, industrial sulfur dioxide, industrial dust, and industrial smoke will appear. By selecting relevant industrial pollution emission data, the entropy method is used to calculate industrial environmental pollution index, which is used as the proxy variable of unexpected output.

Key explanatory variables are government R&D subsidies (SUB) and environmental regulations (ER). Considering the representativeness and availability of data, the logarithm of government R&D subsidies in large- and medium-sized enterprises is used as the proxy variable of R&D subsidy. Furthermore, the proxy variable of environmental regulations adopts the ratio of the actual amount of industrial pollution control investment to industrial added value (Li et al. [30]).

Green innovation is a complex process that involves multiple subjects and multiple factors. Therefore, MGIE is easily affected by the comprehensive influence of many factors. This paper selects enterprise scale, industrial structure, agglomeration, and openness as control variables. (1) Enterprise scale (sca) is generally directly related to R&D funds and the scale effect. The larger the enterprise, the more R&D funds it possesses, that is, more funds can be used to upgrade advanced equipment, which will help improve the resource allocation capacity. However, the larger the enterprise, the more likely it is to rely on existing advantages and lack the motivation to innovate. Moreover, internal mutual prevarication and ineffective management may also occur (Scherer and Ross [49]). In this paper, enterprise scale is measured by the logarithm of the average original value of fixed assets of large- and medium-sized enterprises. (2) Industrial structure (indus). With the development of industrialization, the industrial structure has changed accordingly. The high proportion of the secondary industry indicates that the level of industrialization is heavy. The level of heavy chemical industrialization is closely related to environmental pollution and economic growth. The industrial structure is measured by the proportion of the output value of the secondary industry to gross domestic product (GDP). (3) Agglomeration (agg). Enterprise agglomeration is likely to lead to a competitive effect and stimulate the innovative potential of enterprises constantly. Agglomeration is measured by the logarithm of the number of large- and medium-sized industrial enterprises. (4) Openness (fdi). The impact of openness on MGIE is uncertain. On the one hand, the advanced knowledge and technology brought by foreign direct investment (FDI) can improve the host country’s capacity for green innovation. On the other hand, some high pollution and high emission industries can be transferred to China through FDI, which obviously has a negative effect on MGIE. Openness is measured by the proportion of fixed assets investment funds of large- and medium-sized enterprises from foreign capital. Some variables are processed logarithmically to avoid large differences in the magnitude of the variables and the issue of heteroscedasticity. Information about variable selection is shown in Table 1.

The research area of this paper is 11 provinces (cities) in the Yangtze River Economic Belt of China, including Shanghai, Jiangsu, Zhejiang, Anhui, Hunan, Hubei, Jiangxi, Guizhou, Yunnan, Sichuan, and Chongqing. The data covered the years of 2008–2017, a period of time over which data are available for all variables. Data came from multiple sources, including the China Statistical Yearbook (2009–2018), the China Industrial Statistical Yearbook (2009–2018), and Easy Professional Superior (EPS) database. Descriptive statistics for each variable are shown in Table 2. The standard deviations of human input, financial input, number of domestic invention patents, and sales revenue of new product are up to 107,281, 3,537,871, 6.26 × 10^7^, and 21,861.21, respectively. The above information shows that there are obvious differences in resource endowment and technological output among different regions of the Yangtze River Economic Belt. Although there is a certain gap between the minimum value and the maximum value of resource (energy) input, unexpected output, MGIE, government R&D subsidies, environmental regulations, enterprise scale, industrial structure, agglomeration, and openness, all the standard deviations are relatively small, which means that the dispersion degree is relatively low and the distribution is relatively balanced. The value of MGIE is between 0 and 1, with obvious censored data structure. Therefore, the panel Tobit model is more effective and unbiased under these circumstances.

## 4. Empirical Results and Analysis

### 4.1. Analysis of Estimation Results

According to the results of MGIE in China’s Yangtze River Economic Belt, it can be found that the truncation characteristics are obvious. In order to effectively correct estimated deviation, this paper adopts the panel Tobit model with left truncation set to 0. The estimated results are shown in Table 3. At a significant level of 1%, all test results of model’s χ^2^(chisq) pass the significance test, which shows that the models are reasonable. At the same time, the results of the Hausman test show that the fixed-effect Tobit model is more valid than the random-effect Tobit model. Therefore, this paper mainly analyzes the results of the fixed-effect Tobit model.

First, at a significant level of 5%, we find that government R&D subsidies are conducive to improving MGIE in China’s Yangtze River Economic Belt. In 2017, the average input of government R&D subsidies in the Yangtze River Economic Belt reached 1757.18 million yuan, which was three times the number in 2009. Meanwhile, compared with 2009, the total R&D input, sales revenue of new product, and number of domestic invention patents of industrial enterprises above a designated size increased by 246.93%, 219.03%, and 609.80%, respectively, in 2017. It can be seen that the increase of government R&D subsidies also promotes the innovation input and output of manufacturing enterprises in the Yangtze River Economic Belt. In other words, compared with the crowding-out effect of government R&D subsidies, the promotion effect is more obvious, and ultimately reflects in the fact that government R&D subsidies can improve MGIE.

Secondly, at a significant level of 10%, there is a “U” shaped relationship between environmental regulations and MGIE in the Yangtze River Economic Belt. After calculation, the value of the inflection point in model 2 is 1.047. Additionally, from the descriptive statistics in Table 2, we can see that the average level of environmental regulation is about 1.200, a value that passed the turning point and is located on the right side of the U-shaped curve. That is to say, environmental regulation has a positive role in improving MGIE in the Yangtze River Economic Belt. This outcome is consistent with the findings by Deng et al. [50]. As a matter of fact, since 2015, a series of government documents aiming at strengthening ecological environment protection and green development of manufacturing industry in the Yangtze River Economic Belt have been issued. The themes in these documents include the following: accelerating the green transformation and upgrading of traditional manufacturing industry; strictly control environmental risks of a series of projects along the Yangtze River, such as petroleum processing, chemical raw materials and chemical products production, pharmaceutical production, chemical fiber production, non-ferrous metals, printing and dyeing, paper making, and so on; promote the orderly relocation, transformation, or closure of enterprises with heavy pollution, such as iron and steel, non-ferrous metals, paper making, printing and dyeing, electroplating, chemical active pharmaceutical ingredients (API) manufacturing, chemical industry, and so on; strictly supervise cross-regional transfer of polluting industries; and, lastly, continuously improve the efficiency of resource and energy use and level of cleaner production. Indeed, all of these measures have greatly improved the level of green manufacturing and MGIE in the Yangtze River Economic Belt.

Thirdly, the coefficient of the interactive term between government R&D subsidies and environmental regulation fails to pass the significance test. In other words, government R&D subsidies have no significant positive moderating effect on environmental regulation and MGIE in the Yangtze River Economic Belt. It should be noted that, from the information in the China Procuratorial Statistical Yearbook (2009–2017) and the inspection reports of various provinces in China, the number of cases of corruption, bribery, and malfeasance in the Yangtze River Economic Belt accounted for about 38–40% of the total in China from 2008 to 2016. Under the background of rampant rent-seeking, it is not hard to imagine that, in order to relax the government’s supervision and review, government R&D subsidies are extensively used for rent-seeking, so that the punishment due to environmental regulations is reduced. From this point of view, rent-seeking behavior leads to waste of innovation resources and greatly reduces the effective utilization rate of green innovation input. Therefore, the positive moderating role of government R&D subsidies fails to play to a certain extent.

Fourth, the effects of other control variables on MGIE are as follows: 

(1) Enterprise scale. At a significant level of 5%, the enterprise scale has an inhibiting effect on the improvement of MGIE in the Yangtze River Economic Belt. There are a number of large-scale, state-owned steel, automobile, and chemical enterprises with monopoly characteristics in the Yangtze River Economic Belt. These monopoly enterprises have established market leader status and relatively mature R&D and production management system. The cost or risk of R&D or adoption of green technology will exceed the potential increase of reputation or market share. Therefore, the willingness of these enterprises to carry out green innovation is relatively low.

(2) Industrial structure. At a significant level of 10%, industrial structure has an inhibiting effect on the improvement of MGIE in the Yangtze River Economic Belt. Because the industrialization in the middle and upper reaches of the Yangtze River is still at the stage of accelerating development, heavy chemical industry dominated secondary industry plays a main part in these provinces or cities. The extensive development mode characterized by large consumption of resources has led to the centralized layout of a large number of heavy chemical enterprises in the above areas. The phenomenon of “heavy chemical industry surrounding the river” is obvious. Huge industrial sewage is directly discharged into the Yangtze River without being processed, and a large amount of industrial waste gas is discharged in the production process by some high pollution chemical enterprises, which directly leads to a surge in the unexpected output of green innovation in the Yangtze River Economic Belt and, therefore, a slump of MGIE level.

(3) Agglomeration. At a significant level of 1%, enterprise agglomeration has a positive effect on MGIE in the Yangtze River Economic Belt. Making use of advantages such as location, industry, labor, market, and so on, provinces and cities along the Yangtze River Economic Belt have established five cross regional world-class industrial clusters based on FDI, which are electronic information, high-end equipment, automobile, home appliances, and textile and clothing flusters. These clusters provide possibilities for enterprises within them to adopt environmental protection production technology. At the same time, the positive externalities brought about by them can also promote technological progress and expansion of environmental protection technology. As a result, the “Pollution Halo Hypothesis” is reduced and MGIE is improved.

(4) Openness. Openness does not have any statistical impact on MGIE in the Yangtze River Economic Belt. One possible reason is that the human cost of the Yangtze River Economic Belt is increasing year by year. By 2017, the demographic dividend effect of the Yangtze River Economic Belt has gradually disappeared. The total wages of China’s urban employment reached 5,550,817 million yuan, an increase of 309.94% since 2008. In comparison, Laos, Vietnam, Cambodia, and other developing countries adjacent to the upper reaches of the Yangtze River Economic Belt have a greater comparative advantage in human cost. Therefore, some foreign enterprises in the Yangtze River Economic Belt began to shift their investment destination to Southeast Asian countries, which finally leads to an unobvious effect of openness to the improvement of MGIE in Yangtze River Economic Belt.

### 4.2. Analysis of Endogenous Problems

The two-way causal relationship between government R&D subsidies, environmental regulation, and MGIE will cause endogenous problems. Such problems are solved by using the instrumental variable method, and constructing the panel Ivtobit model. Generally speaking, the ideal instrumental variable is selected from the historical or geographical aspect, so that a direct correlation with endogenous variables can be maintained, and exogenous requirements can be met. Following this principle, together with the consideration of the correlation between fiscal revenue and R&D subsidies, and the non-correlation relationship between fiscal revenue and MGIE, the negative correlation between environmental regulation and the air circulation coefficient, as well as natural phenomena attributing to satisfying the requirements of exogenesis, this paper selects fiscal revenue and the air circulation coefficient as instrumental variables of government R&D subsidies and environmental regulations, respectively (Shen [51]; Shi and Xu [52]). As shown in Table 4, each instrumental variable passes the weak instrumental variable test and exogenous test, which indicates that the selection of instrumental variable is effective. At a significant level of 5%, government R&D subsidies have a positive effect on MGIE, and the positive effect gets stronger. More explicitly, ignoring endogenous problems will underestimate the positive effect of government R&D subsidies on MGIE.

### 4.3. Robustness Test

The paper firstly replaced the core indicators of government R&D subsidies with its alternatives, and a robustness test is done to the results from the perspective of variables. The proportion of government technology subsidies to the main business income of the enterprise is selected as an alternative indicator of government R&D subsidies. Table 5 demonstrates the estimation results of the panel Tobit model after adjusting the core indicator with the above alternative. At a significant level of 5%, the government R&D subsidies can promote MGIE in the Yangtze River Economic Belt. At a significant level of 10%, there is a “U-shaped” relationship between environmental regulations and MGIE. Additionally, the positive moderating effect of government R&D subsidies between environmental regulations and MGIE fails to pass the significance test. As compared with the estimation results in Table 3, it is clear that the estimation results are essentially the same. As a result, we are confident that the empirical outcomes in Table 3 are robust and reliable.

Secondly, it may take a long time for the government R&D subsidies and environmental regulations on MGIE to perform. Therefore, the lag of government R&D subsidies and environmental regulations is taken into the model again to test whether the effects are consistent in the long term and the short term. The specific results are shown in Table 6. We can find that, at a significance level of 10%, the government R&D subsidies can promote MGIE in the Yangtze River Economic Belt. The coefficient of the interactive term between government R&D subsidies and environmental regulations fails to pass the significance test. Besides, affected by the selected time limit, the “U-shaped” relationship between environmental regulations and MGIE also fails to pass the significance test. However, generally speaking, the overall results are basically consistent with Table 3.

## 5. Conclusion and Policy Implications

By using the SBM-DEA model with consideration of unexpected outputs, this paper measures the level of MGIE in the Yangtze River Economic Belt. With the introduction of the interactive term between government subsidies and environmental regulations and the quadratic term of environmental regulations into the model, the panel Tobit model is further used to empirically test the effects of government R&D subsidies and environmental regulations on MGIE in the Yangtze River Economic Belt. As evidenced from the empirical results, first, government R&D subsidies are conducive to improving MGIE in the Yangtze River Economic Belt. Government R&D subsidies and the economic signals released can provide other external financing opportunities, which is conducive to alleviating the problem of insufficient funds as well as reducing the cost and risk of green technology R&D activities. Secondly, there is a positive “U-shaped” relationship between environmental regulations and MGIE in the Yangtze River Economic Belt, and the level of environmental regulations has passed the inflection point and is on the right side of the U-shaped curve. Because the “innovation compensation effect” exceeds the “follow-the-cost effect” at this point, environmental regulations have a positive role in improving MGIE in the Yangtze River economic belt. Thirdly, the coefficient of the interactive term between government R&D subsidies and environmental regulations fails to pass the significance test. More explicitly, government R&D subsidies fail to effectively stimulate the “innovation compensation effect” of environmental regulation, which indicates that the positive moderating role of government R&D subsidies fails to play its role. Lastly, the characteristics of spatial layout, where a large number of monopoly enterprises agglomerating along the Yangtze River Economic Belt, and the industry structure of heavy chemical enterprise are not conducive to the improvement of MGIE. On the contrary, agglomeration has a significant positive role in improving MGIE in the Yangtze River Economic Belt. In addition, the degree of openness fails to pass the significance test.

The policy implications of this paper are as follows. First, in view of the significant positive effect of government R&D subsidies on MGIE in the Yangtze River Economic Belt, government should guide manufacturing enterprises to increase green innovation input by increasing government R&D subsidies. However, attention should be paid to the study of new methods, so as to avoid the “crowding out effect” and attract more manufacturing enterprises to engage in green technology activities. Second, considering the severe pressure of resources and environment along the Yangtze River Economic Belt, it is still necessary to highlight the important role of environmental regulations in improving MGIE in the near future. Through the establishment of a variety of environmental policies tools that are of command type, market-driven, or social willingness, provinces and cities along the Yangtze River Economic Belt can be guided to develop green manufacturing, advanced manufacturing, and intelligent manufacturing, and further reduce the unexpected output of manufacturing industry. Third, in order to exert the positive moderating role of government R&D subsidies, an optional path is to strengthen the honest construction of government departments along the Yangtze River Economic Belt. This can improve the transparency of government R&D subsidies, and reduce the reverse seeking subsidies of manufacturing enterprises and corruption of government. To ensure the effectiveness of the use of government R&D subsidies and the implementation of environmental regulations, it is important to strengthen the supervision of environmental regulations and take environmental quality as an important indicator of government performance evaluation. Fourth, in terms of control variables, in order to reduce the negative effects of the enterprise scale and industrial structure on MGIE in the Yangtze River Economic Belt, it is necessary to cultivate small- and medium-sized scientific and technological enterprises and promote the marketization reform of large state-owned enterprises with a monopoly characteristic. In the meanwhile, for the purpose of establishing a rational and high-end industry structure of the Yangtze River Economic Belt, attention should be paid to the new technologies, new products, new formats, and novel business models, and efforts should be made to realize the service-oriented, high-end, intelligent, knowledgeable, and low-carbon industry.

## 6. Discussion

At present, China is committed to building the Yangtze River Economic Belt into a world-famous manufacturing economic belt, similar to the Mississippi River in the United States and the Rhine River in Germany. However, the manufacturing industry along the Yangtze River Economic Belt is facing severe resource and environmental constraints. In order to improve the development quality of manufacturing industry, measures should be taken to improve the expected output of invention patents and develop new products through green innovation, and to reduce the unexpected output of environmental pollution. As an important participant, government intervention will directly or indirectly affect MGIE. In particular, government R&D subsidies and environmental regulations are typical forms. A joint analysis of multiple policies is one of the hot topics of future policy research. The main contributions of this paper is that government R&D subsidies, environmental regulations, and MGIE along the Yangtze River economic belt are included in a unified analyzing framework. The nonlinear characteristics of environmental regulation and the moderating effect of government R&D subsidies are considered comprehensively. The relationship between government R&D subsidies, environmental regulations, and MGIE in the Yangtze River Economic Belt is analyzed.

Nevertheless, this paper has several limitations that can be addressed in future studies. On the one hand, this paper does not consider the fact that there is obvious spatial correlation and interdependence of green innovation, the flow of factors, and environmental pollution among regions. Therefore, it is necessary to include spatial factor as one of the influencing factors of MGIE. In fact, there exist more and more innovation contacts between provinces, and the innovation network is becoming increasingly intensified [53]. On the other hand, it should be noted that the selection of indicators plays a key role in the conclusions. Even though the indicators have been replaced in the robustness test, there may still be some deficiencies. For example, the actual amount of industrial pollution control completed investment, frequencies of supervision and inspection, government environmental protection expenditure, emission fee/tax, and so on can all be used to indicate environmental regulations. One question that needs further discussion is whether the conclusions will change when other variables are selected for regression.

## Figures and Tables

**Table 1:** Variable selection and processing.

	Name		Indicator	Unit
variables about efficiency calculation	input variables	human input	the full-time equivalent of R&D personnel in industrial enterprises above designated size	person-year
		financial input	the R&D expenditure in industrial enterprises above designated size	ten thousand yuan
		resources (energy) input	the energy consumption per unit of industrial production	ten thousand yuan/ton of standard coal
	output variables	expected output	the number of domestic invention patents	pcs
			sales revenue of new product	ten thousand yuan
		unexpected output	industrial environmental pollution index	
variables about effect analysis	explanatory variables	government R&D subsidies	the logarithm of the government funds for science and technology activities of large- and medium-sized industrial enterprises	
		environmental regulation	the ratio of the actual amount of industrial pollution control completed investment to industrial added value	%
	control variables	enterprises scale	the logarithm of the average original value of fixed assets of large- and medium-sized enterprises	ten thousand yuan
		industrial structure	the proportion of the output value of the secondary industry to gross domestic product (GDP)	1
		agglomeration	the logarithm of the number of large- and medium-sized industrial enterprises	
		openness	the proportion of fixed assets investment funds of large- and medium-sized enterprises from foreign capital	%

**Table 2:** Descriptive statistics of variables.

Variable	Obs	Mean	SD	Min	Max
human input	110	92,871.16	107,281	6134.28	455,468
financial input	110	3094,582	3537,871	77,081.4	1.80 × 10^7^
resource(energy) input	110	0.662	0.483	0.193	2.277
the number of domestic invention patents	110	5.55 × 10^7^	6.26 × 10^7^	615,675	2.90 × 10^8^
sales revenue of new product	110	15,675.74	21,861.21	279	140,346
unexpected output	110	0.034	0.003	0.028	0.042
MGIE	110	0.731	0.301	0.093	1
government R&D subsidies	110	11.348	0.846	9.060	12.825
environmental regulations	110	1.200	0.468	0.520	2.660
enterprises scale	110	9.464	0.732	7.873	11.271
industrial structure	110	0.462	0.056	0.298	0.554
agglomeration	110	9.385	0.852	7.753	11.090
openness	110	0.011	0.013	0.000	0.084

**Table 3:** Estimation results of effects of government R&D subsidies and environmental regulations on MGIE.

Variable	Model 1	Model 2	Model 3	Model 4
*SUB*	0.211 ** (2.34)		0.147 (1.53)	0.088 (0.85)
*ER*		−0.888 * (−1.71)	−0.768 (−1.27)	−1.399 ** (−1.98)
*ER2*		0.424 ** (2.04)		0.376 (1.49)
*SUB∗ER*			0.372(1.57)	0.055 (0.95)
*sca*	−0.401 ** (−2.08)	−0.273 ** (−2.25)	−0.400 *** (−2.47)	−0.404 ** (−2.43)
*indus*	−2.380 * (−1.95)	−4.396 *** (−4.11)	−3.001 ** (−2.51)	−4.175 *** (−4.36)
*agg*	0.495 ** *(2.82)	0.639 *** (5.00)	0.580 *** (5.18)	0.575 *** (4.90)
*fdi*	0.543 (0.10)	3.459 (0.55)	3.737 (0.75)	3.723 (0.72)
χ^2^	112.71 *** (*p* = 0.0000)	169.51 ***(*p* = 0.0000)	271.96 *** (*p* = 0.0000)	252.77 *** (*p* = 0.0000)

Note: Figures in parentheses are *t* values; * *p* < 0.1, ** *p* < 0.05, *** *p* < 0.01.

**Table 4:** Estimation results of the Ivtobit model of the effects of government R&D subsidies and environmental regulations on MGIE.

Variable	Model 1	Model 2	Model 3
*SUB*	0.540 *** (4.43)		0.633 ** (2.29)
*ER*		−4.396 (−1.22)	0.052 (0.01)
*ER^2^*		1.521 (1.12)	−0.092 (−0.06)
*Sca*	−0.544 *** (−4.08)	0.069 (0.47)	−0.615 * (−1.76)
*Indus*	−0.791 (−1.19)	−1.543 (−0.62)	0.462 (0.18)
*Agg*	0.237 *** (3.01)	0.193 (1.52)	0.196 ** (1.98)
*Fdi*	1.656 (0.601)	4.057 (0.75)	0.980 (0.20)
con_s	−2.128 *** (3.63)	1.684 (0.77)	−2.614 (−0.82)
χ^2^	59.14 *** (*p* = 0.0001)	21.35 *** (*p* = 0.0016)	48.28 *** (*p* = 0.0000)
Wald test	33.55 *** (*p* = 0.0000)	12.04 *** (*p* = 0.0024)	52.48 *** (*p* = 0.0024)
AR	138.57 *** (*p* = 0.0000)	83.05 *** (*p* = 0.0000)	163.97 *** (*p* = 0.0024)

Note: Figures in parentheses are *t* values; * *p* < 0.1, ** *p* < 0.05, *** *p* < 0.01.

**Table 5:** Estimation results of the effects of government R&D subsidies and environmental regulations on MGIE (from variable perspective).

Variable	Model 1	Model 2	Model 3	Model 4
*SUB*	0.062 ** (2.12)		0.042 (1.59)	0.034 (1.07)
*ER*		−0.888 * (−1.71)	−0.729 * (−1.82)	−1.557 ** (−2.51)
*ER2*		0.424 ** (2.04)	0.351 ** (2.17)	0.346 ** (2.20)
*SUB∗ER*				0.074 (1.13)
*sca*	−0.177 (−1.46)	−0.273 ** (2.25)	−0.267 *** (−2.87)	−0.340 *** (−2.78)
*indus*	−0.905 (−0.97)	−4.396 *** (−4.11)	−3.320 ** (−2.53)	−3.342 ** (−2.53)
*agg*	0.353 ** (1.96)	0.639 *** (5.00)	0.517 *** (3.79)	0.501 *** (3.69)
*fdi*	−1.136 (−0.26)	3.459 (0.55)	1.783 (0.39)	2.111 (0.47)
χ^2^	110.86 (*p* = 0.0000)	169.51 (*p* = 0.0000)	524.57 *** (*p* = 0.0000)	628.35 *** (*p* = 0.0000)

Note: Figures in parentheses are *t* values; * *p* < 0.1, ** *p* < 0.05, *** *p* < 0.01.

**Table 6:** Estimation results of the effects of government R&D subsidies and environmental regulations on MGIE (from a time perspective).

Variable	Model 1	Model 2	Model 3	Model 4
*L.SUB*	0.208 ** (2.12)		0.143 * (1.76)	0.167 (1.21)
*L.ER*		0.00004 (0.01)	0.003 (0.78)	0.003 (0.81)
*L.ER2*		0.0110 *** (2.93)	0.096 *** (4.25)	0.159 (0.55)
*L.SUB∗L.ER*				−0.016 (0.24)
*sca*	−0.311 (−2.08)	−0.255 (−1.29)	−0.339 ** (−2.11)	−0.336 ** (−1.98)
*indus*	−2.529 ** (−2.12)	−4.459 *** (−3.76)	−3.729 *** (−3.14)	−3.730 *** (−3.26)
*agg*	0.370 ** (2.44)	0.560 *** (2.97)	0.474 ** (2.39)	0.469 ** (2.35)
*fdi*	11.385 ** (−2.19)	13.086 (1.11)	12.796 (1.54)	13.309 (1.43)
χ^2^	111.68 *** (*p* = 0.0000)	134.75 *** (*p* = 0.0000)	448.64 *** (*p* = 0.0000)	640.12 *** (*p* = 0.0000)

Note: Figures in parentheses are *t* values; * *p* < 0.1, ** *p* < 0.05, *** *p* < 0.01.
